# Supplementary material for: Identification and functional analysis of non-coding regulatory small RNA FenSr3 in Bacillus amyloliquefaciens LPB-18
Source: PeerJ. 2023 May 15;11:e15236. doi: 10.7717/peerj.15236 (PMC10194069; doi:10.7717/peerj.15236)
Supplement: Supplemental Information 4 [file peerj-11-15236-s004.zip › KO/CK-vs-T1_map/map00909.html]

KEGG PATHWAY: Sesquiterpenoid and triterpenoid biosynthesis - Reference pathway


|  |  |
| --- | --- |
| **Sesquiterpenoid and triterpenoid biosynthesis - Reference pathway** |  |

[
Pathway menu
| Organism menu
| Pathway entry
| Show description
| User data mapping
]

Sesquiterpenoids (C15 terpenoids) are a group of terpenoids consisting of three isoprene units. They are derive from farnesyl diphosphate (FPP) and can be cyclized to produce various skeletal structures. Sesquiterpenoid biosynthesis begins with the loss of diphosphate from FPP under the action of sesquiterpene synthesis enzymes, generating an allylic cation that is highly susceptible to intramolecular attacks. Cyclization of the farnesyl cation may take place onto either of the remaining double bonds with the result that 6-, 10-, or 11-membered rings may be formed. Many sesquiterpenoids have been isolated from plants, fungi, marine organisms, and Streptomyces species. This map shows a few examples of acyclic and cyclic sesquiterpenoids.

|  |  |  |
| --- | --- | --- |
| Reference pathway | 184% 150% 122% 100% 82% 67% 55% | 图片下载 |
